# Cell cycle- and dose-dependent effects on mitochondrial DNA copy number variation following irradiation

**DOI:** 10.1242/jcs.263642

**Published:** 2025-08-08

**Authors:** Ryosuke Seino, Kai Nishikubo, Hisanori Fukunaga

**Affiliations:** Department of Biomedical Science and Engineering, Faculty of Health Sciences, Hokkaido University, Sapporo 060-0812, Japan

**Keywords:** Cell cycle, DNA copy number variation, FUCCI, Mitochondrial DNA, mtDNA, Radiation

## Abstract

Cell survival after irradiation depends on the cell cycle at the time of exposure. This has been thought to be due to cell cycle-dependent nuclear DNA damage repair mechanisms. Here, we show the relationships between the exposed dose, the cell cycle phase at the time of exposure and changes in mitochondrial DNA copy numbers (mtDNAcn) after irradiation. We used a fluorescent ubiquitylation-based cell cycle indicator (FUCCI), which allows visualization of the cell cycle, and confirmed cell cycle synchronization in human cervical HeLa cells. In synchronous HeLa-FUCCI cells, the mtDNAcn changed with the progression of the cell cycle. Also, G1 phase-synchronized cells showed a dose-dependent increase of mtDNAcn at 48 h after X-ray exposure, whereas G2 cells showed a dose-dependent increase at 24 h. In addition, S phase-synchronized cells showed a dose-dependent increase at 24 and 48 h after irradiation. These results showed the cell cycle- and dose-dependent effects on mtDNAcn after irradiation, which might shed light on the emerging role of mitochondrial genome and in cell survival.

## Teaser

**Summary:** The mitochondrial DNA copy number (mtDNAcn) fluctuates in a cell cycle-dependent manner and the mtDNAcn after irradiation depends on the cell cycle phase at the time of exposure and the exposed dose.

## INTRODUCTION

Since the historic discovery by Terasima and Tolmach in the 1960s that cell survival after irradiation depends on the cell cycle phase at the time of exposure (Terasima and Tolmach, 1961, 1963a), several studies have been conducted on the mechanisms of DNA damage repair during each cell cycle phase (Hustedt and Durocher, 2016; Pawlik and Keyomarsi, 2004; Tamulevicius et al., 2007). Most cells are sensitive in the G2 and M phases, less sensitive in the G1 phase and least sensitive in the late S phase (Seino et al., 2024) because different DNA damage repair mechanisms are thought to be activated during specific phases of the cell cycle (Escribano-Díaz et al., 2013). DNA double strand breaks can be repaired via a homologous recombination-dependent mechanism during the G2/M phases of the cell cycle, whereas non-homologous end joining mechanisms are activated during G1/G0; however, these are findings concerning nuclear DNA (nDNA). The underlying mechanisms of cytoplasmic DNA damage repair and maintenance at each cell cycle phase remain to be determined.

Human mitochondrial DNA (mtDNA) is a 16,569 base-pair cyclic multi-copy genome (Anderson et al., 1981), usually with tens to thousands of copies per cell. In eukaryotic cells, both nDNA and mtDNA cooperate to regulate cellular functions (St John, 2019). Compared to nDNA, mtDNA is a small genome, but mRNA transcribed from mtDNA accounts for ∼30% of all cellular mRNA in the heart and 5–25% in other organs (Mercer et al., 2011). Point mutations, deletions and copy number reductions in mtDNA have been implicated in mitochondrial dysfunction. Thus, the proper control of mtDNA copy numbers (mtDNAcn) is of importance not only at the cellular level but also at the whole-organism level (Clay Montier et al., 2009). In fact, as a potential biomarker, the mtDNAcn in the peripheral blood reflects metabolic health across multiple tissues (Yang et al., 2021) and cord blood mtDNAcn correlates with birth outcomes (Fukunaga and Ikeda, 2023).

**Fig. 1.**
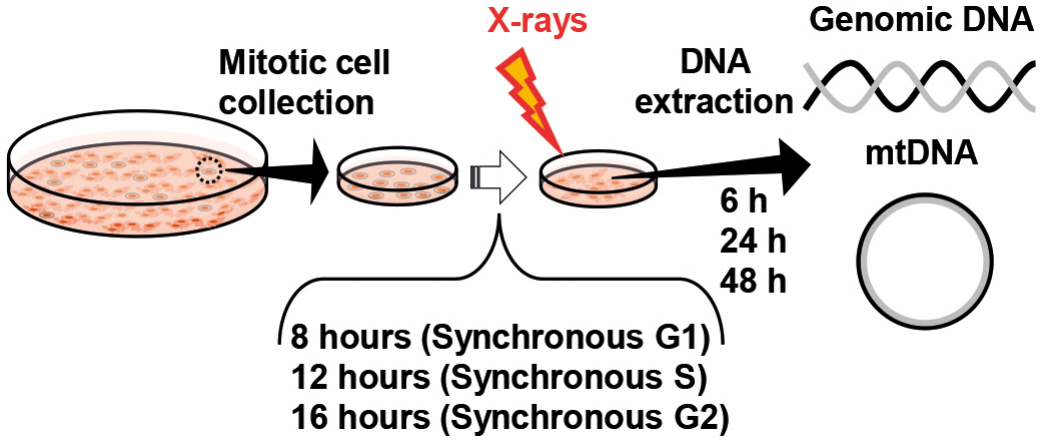
**Overview of the experimental methods.** Mitotic cells were collected, and cultured for 8, 12 and 16 h to synchronize them into G1, S and G2 phases, respectively. Then, X-ray irradiation was performed, and DNA was extracted at 6, 24 and 48 h after irradiation.

In general, radiation-damaged cells are known to synthesize their mtDNA (Kam and Banati, 2013). However, the correlation between the mtDNAcn and the cell cycle phase at the time of exposure to radiation and its dose dependence remains to be determined. The purpose of this study is to elucidate whether the radiation response to mtDNAcn is dependent on the phase of the cell cycle at the time of exposure. We used florescent ubiquitylation-based cell cycle indicators (FUCCI) (Sakaue-Sawano et al., 2008), which can visualize the G1/G0 and S/G2/M phases at different fluorescence wavelengths by labeling geminin expressed in S/G2/M phase and Cdt1 expressed in the G1 phase with green and red fluorescent proteins, respectively. In this study, we synchronized the HeLa-FUCCI cells, a cervical cancer cell line expressing FUCCI, using the mitotic harvesting method as a cell cycle synchronization approach (Seino et al., 2023; Terasima and Tolmach, 1963b). Then, HeLa-FUCCI cells in the synchronous G1, G2, and S phases were irradiated with 0, 0.5, 2, 4 or 8 Gy of X-rays and chronological changes in the mtDNA-to-nDNA ratio (relative mtDNAcn) after irradiation were measured using real-time quantitative PCR (Fig. 1).

In synchronous HeLa-FUCCI cells, we found that the mtDNAcn fluctuates with cell cycle progression. Following X-ray exposure, mtDNAcn exhibited a dose-dependent increase, with the peak response occurring at 48 h post-irradiation in G1 phase cells, at 24 h in G2 phase cells, and at both 24 and 48 h in S phase cells. These findings provide new insights into the emerging role of the mitochondrial genome in cell cycle-dependent radiosensitivity.

## RESULTS

### Cyclic changes of relative mtDNAcn in synchronous HeLa-FUCCI cells

**Fig. 2.**
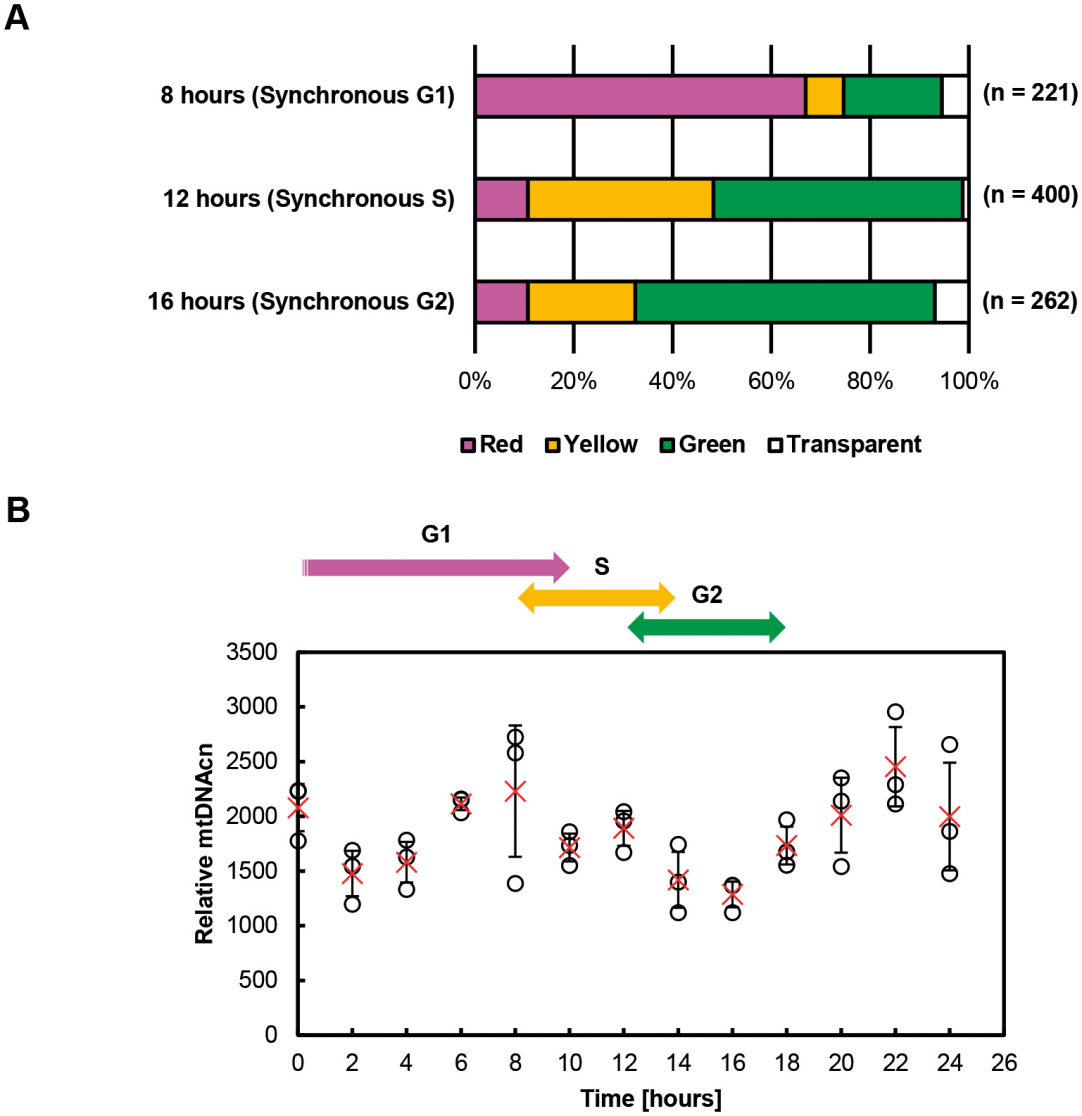
**Time-dependent changes in fluorescence and relative mtDNAcn in synchronous HeLa-FUCCI cells.** (A) Summary graph of nuclear fluorescent color distributions in synchronous HeLa-FUCCI cells. Although the FUCCI system does not provide exact phase separation, the fluorescence pattern combined with timing after synchronization provides a reliable approximation of cell cycle enrichment. Therefore, the time points designated as ‘synchronous S’ and ‘synchronous G2’ reflect relative maxima in S and G2 cell populations, respectively, rather than pure phase populations. Results are for *n*=3 independent experiments.  (B) Time-dependent changes in relative mtDNAcn in synchronous HeLa-FUCCI cells. Each data point is shown as a circle, and the mean is shown as a red cross. Error bars indicate s.d. (*n*=3).

Fig. 2A shows the fluorescent color distributions in synchronous HeLa–FUCCI cells 8, 12 and 16 h after cell cycle synchronization. Approximately 67% of the cells showed red fluorescence at 8 h (synchronous G1 phase), whereas 61% showed green at 16 h (synchronous G2 phase). At 12 h, 37.5% cells showed yellow (early S phase) and 50.5% showed green fluorescence (S/G2 phase). The observed color patterns closely reproduce the findings of previous studies (Seino et al., 2023, 2024; Terasima and Tolmach, 1961). In addition to fluorescence-based color classification, flow cytometric analysis demonstrated that the cell cycle was moderately synchronized at each time point. This approach is commonly used in studies employing FUCCI reporters, where precise phase separation is limited but relative enrichment is sufficient to study cell cycle-dependent trends.

Fig. 2B shows the time-dependent changes in relative mtDNAcn in synchronous HeLa-FUCCI cells. The synchronous cells were considered to have entered G1 phase at 8 h after mitotic collection, S phase at 10–12 h, and G2 phase at 14–16 h, based on previous studies (Seino et al., 2024). Interestingly, in this study, the relative mtDNAcn increased in the G1 phase, decreased in the S phase and increased in the G2 phase. Then, the ratio returned to the original level after approximately one iteration of the cell cycle.

### Post-exposure relative mtDNAcn changes in the G1, G2 and S phase-synchronized cells

**Fig. 3.**
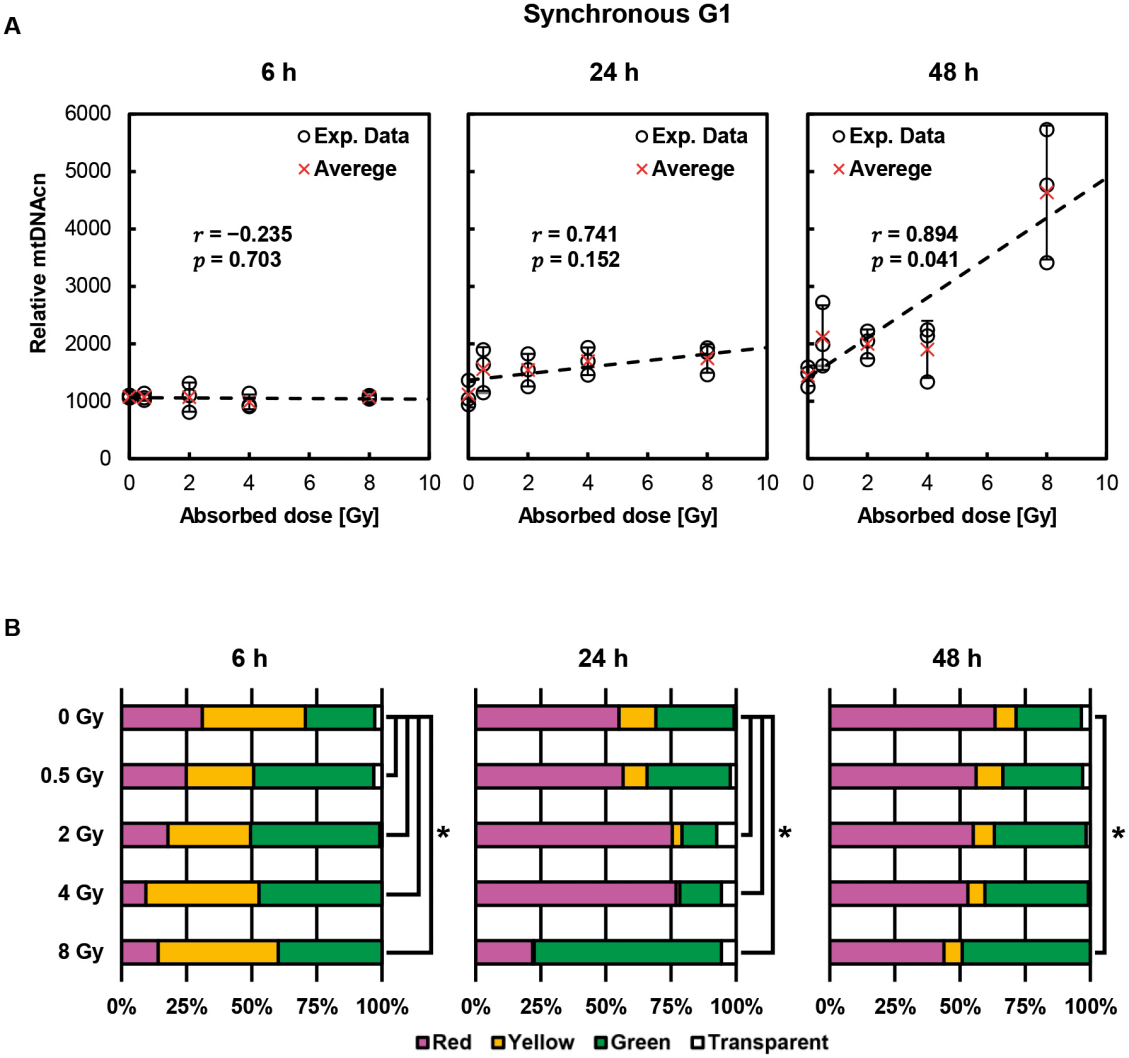
**Post-irradiation changes in relative mtDNAcn and nuclear fluorescence percentages in cells synchronized in G1 phase at the time of exposure.** (A) Time-dependent changes in relative mtDNAcn in cells synchronized in G1 phase. Each data point is shown as a circle, and the mean is shown as a red cross. Error bars indicate s.d. (*n*=3). Pearson's correlation coefficient analysis revealed a significant correlation between absorbed dose and mtDNAcn 48 h after irradiation. A statistically significant correlation was observed when *P*<0.05. (B) Percentage fluorescence in the cell nuclei after exposure. Results are for *n*=3 independent experiments.  Significance was defined as *P<0.05 (Chi-squared test).

Post-irradiation alterations in relative mtDNAcn in G1-synchronized cells are illustrated in Fig. 3A. The irradiated cells generally showed an increase in the relative mtDNAcn with incubation time, compared to the non-irradiated cells, regardless of the X-ray exposure dose. At 48 h after irradiation, a significant dose dependence of relative mtDNAcn was observed (*P=*0.041). Fig. 3B shows changes in the fluorescent color distribution of the cell nuclei after irradiation. At 6 h post exposure, irradiated cells showed dose-independent changes, whereas at 24 h, significant changes were observed in cells irradiated with 2, 4 and 8 Gy. Notably, the percentage of green cells in the 8 Gy-irradiated group was higher, suggesting G2 arrest. Interestingly, only the 8 Gy-irradiated group showed a marked increase in relative mtDNAcn 48 h after exposure. However, there was no apparent correlation between these color percentages and relative mtDNAcn, which means a relationship between irradiation-induced cell cycle delay and mtDNAcn is not supported.

**Fig. 4.**
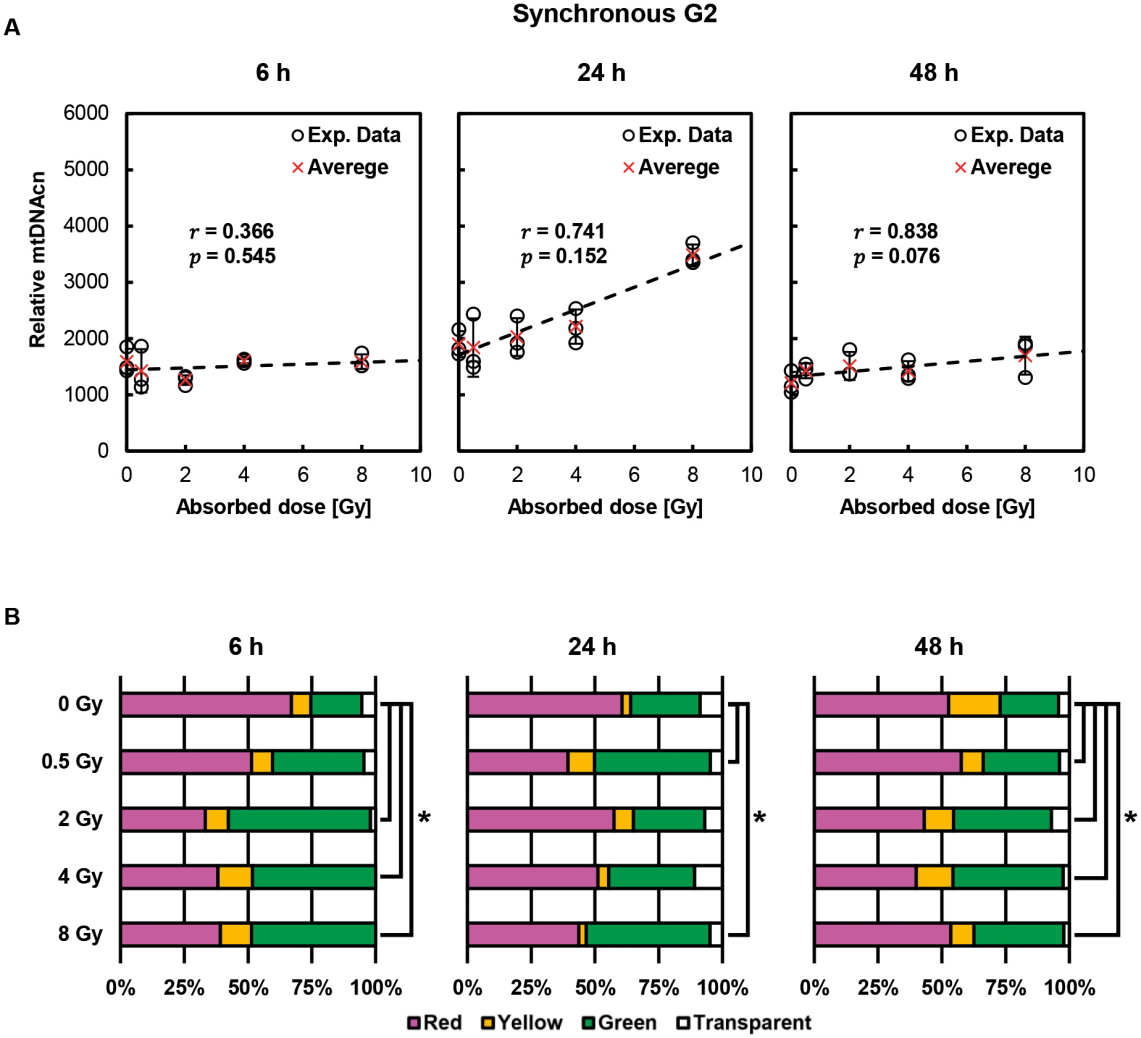
**Post-irradiation changes in relative mtDNAcn and nuclear fluorescence percentages in cells synchronized in G2 phase at the time of exposure.** (A) Time-dependent changes in relative mtDNAcn in cells synchronized in G2 phase. Each data point is shown as a circle, and the average is shown as a red cross. Error bars indicate s.d. (*n*=3). Pearson's correlation coefficient analysis revealed a significant correlation between absorbed dose and mtDNAcn 24 h after irradiation. A statistically significant correlation was observed when *P*<0.05. (B) Percentage fluorescence in the cell nuclei after exposure. Results are for *n*=3 independent experiments. Significance was defined as *P<0.05 (Chi-squared test).

Fig. 4A depicts post-irradiation alterations in relative mtDNAcn in cells synchronized in G2 phase at the time of exposure. Although the increase in relative mtDNAcn at 24 h after irradiation was significantly dose dependent (*P=*0.011), there was no increase at 6 and 48 h after exposure. Thus, synchronous G1 and G2 cells showed distinctly different responses. Fig. 4B shows changes in the fluorescent color distribution of the cell nuclei after irradiation. At 6 h after irradiation, only cells irradiated with 2 Gy or more showed a significant increase in green cells, indicating potential G2 arrest. At 24 h, significant changes were observed in the 0.5 Gy and 8 Gy groups. However, there was no apparent correlation between these color percentages and relative mtDNAcn, and hence these data do not support the idea that there is a relationship between irradiation-induced cell cycle delay and mtDNAcn.

**Fig. 5.**
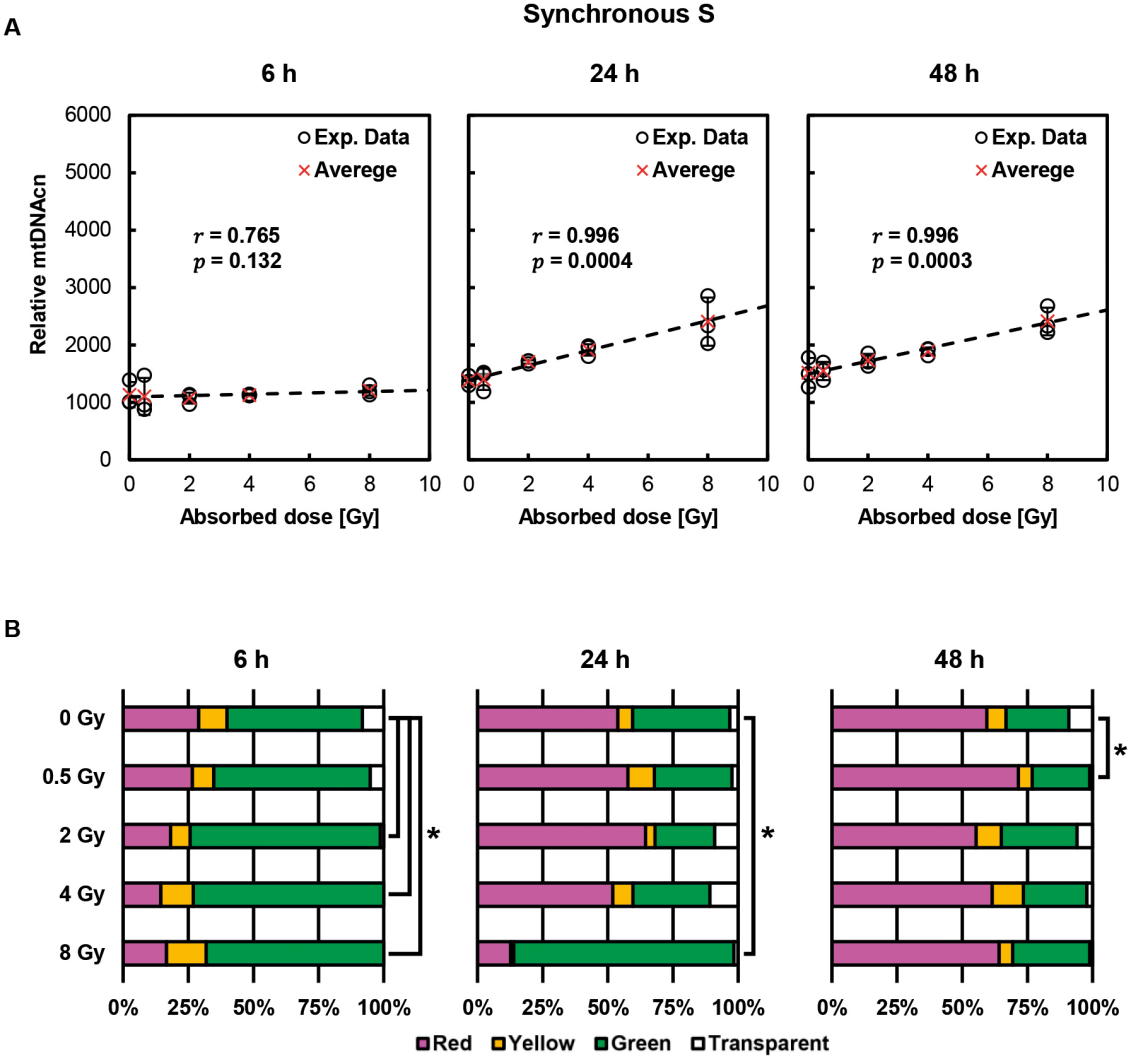
**Post-irradiation changes in relative mtDNAcn and nuclear fluorescence percentages in cells synchronized in S phase at the time of exposure.** (A) Time-dependent changes in relative mtDNAcn in cells synchronized in S phase. Each data point is shown as a circle, and the average is shown as a red cross. Error bars indicate s.d. (*n*=3). Pearson's correlation coefficient analysis revealed a significant correlation between absorbed dose and mtDNAcn 24 and 48 h after irradiation. A statistically significant correlation was observed when *P*<0.05. (B) Percentage fluorescence in the cell nuclei after exposure. Results are for *n*=3 independent experiments. Significance was defined as *P*<0.05 (Chi-squared test).

Fig. 5A shows post-irradiation alterations in relative mtDNAcn in cells synchronized in S phase at the time of exposure. At 24 and 48 h after irradiation, the increase in the relative mtDNAcn showed a significant dose dependence (*P=*0.0004 and 0.0003, respectively), whereas no increase was observed at 6 h post exposure. Thus, interestingly, this indicates intermediate characteristics between G1 and G2 phase cells. Fig. 5B shows changes in the fluorescent color distribution of the cell nuclei after irradiation. At 6 h post exposure, a significant increase in green cells was observed in the ≥2 Gy-irradiated groups. At 48 h, only the 0.5 Gy group showed a significant change. There was no apparent correlation between these color percentages and relative mtDNAcn, so a relationship between irradiation-induced cell cycle delay and mtDNAcn is not supported.

### Post-exposure relative mtDNAcn changes with etoposide treatment

To assess whether synchronization could be further optimized, we employed etoposide, an anticancer agent (Hande, 1998), for cell cycle arrest. In HeLa-FUCCI cells treated with 1 μM etoposide for 24 h, the proportion of green nuclei significantly increased, indicating effective G2 phase arrest (Fig. S2A). The surviving fraction after X-ray irradiation was comparable to that of untreated controls (Fig. S2B), consistent with a previous report (Minehan and Bonner, 1993). However, the relative mtDNAcn increased at 6 h post irradiation and subsequently decreased from 24 to 48 h, regardless of dose (Fig. S2C). The distribution of nuclear fluorescence revealed that most cell nuclei were green at 6 h across all dose groups, with increasing proportions of red and yellow nuclei observed from 24 to 48 h (Fig. S2D). Notably, no clear correlation was observed between the distribution of fluorescence colors and relative mtDNAcn.

## DISCUSSION

### Cell cycle- and dose-dependent effects on mtDNAcn following irradiation

In the present study, using HeLa-FUCCI cells, we showed that the relative mtDNAcn in the non-irradiated cells fluctuates in a cell cycle-dependent manner. This suggests that mtDNA is continuously synthesized at a rate that generally exceeds the rate of degradation, except during S phase when nuclear DNA replication leads to a relative decrease. Thus, the relative copy numbers per cell could remain constant at some level, even when cytoplasmic division occurs in the M phase.

**Fig. 6.**
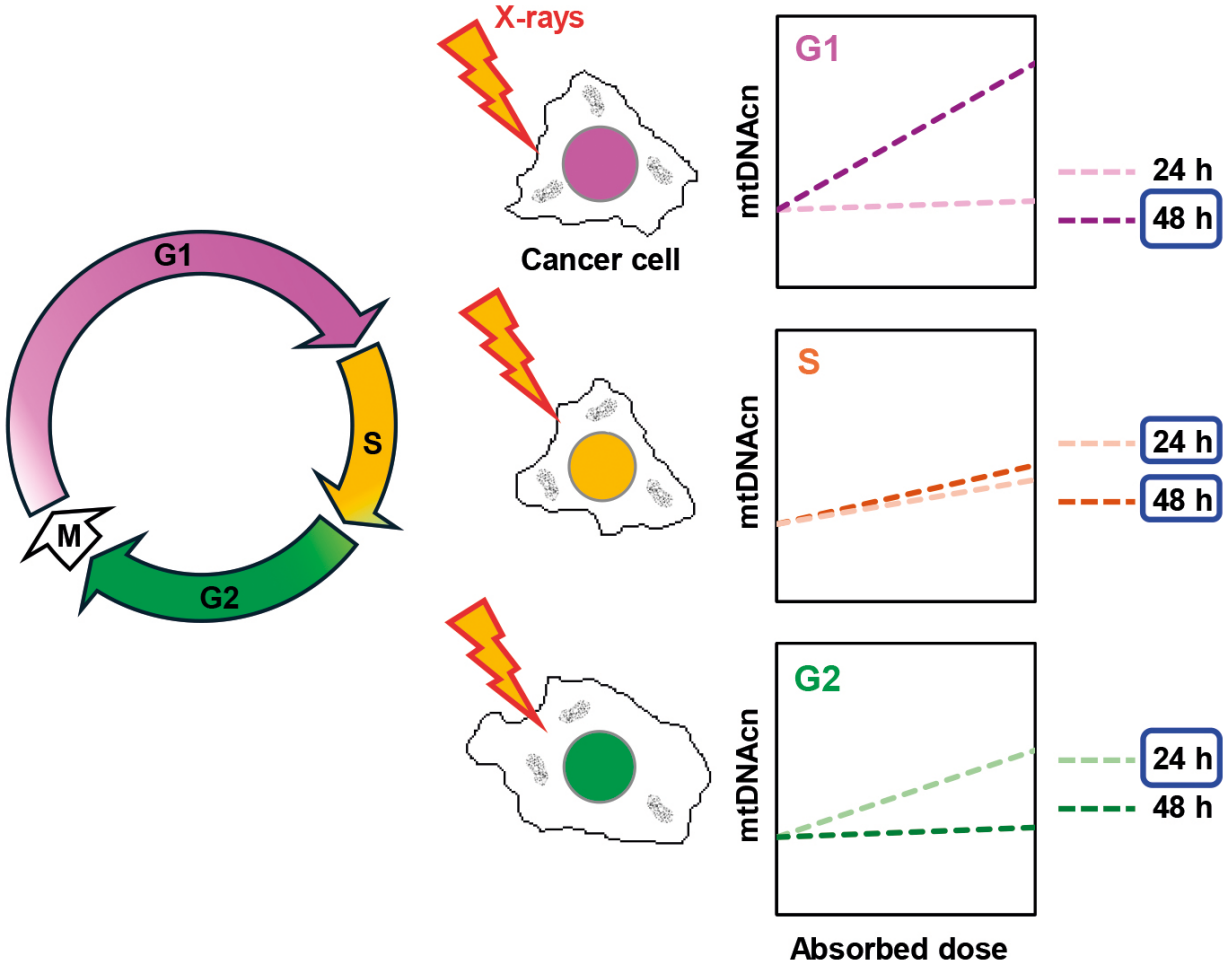
**Cell cycle- and dose-dependence of relative mtDNAcn increase after irradiation.** G1 phase-synchronized cells showed dose-dependence in the relative mtDNAcn increase at 48 h after X-ray exposure, G2 cells at 24 h, and S phase cells at 24 and 48 h.

We also demonstrated that the dose dependence of relative mtDNAcn increase after X-ray exposure depends on the cell cycle phase at the time of exposure: G1 phase-synchronized cells showed a dose-dependence of mtDNA increase at 48 h after exposure, G2 cells at 24 h, and S phase cells at 24 and 48 h (Fig. 6). In the 8 Gy-irradiated group, the proportion of green cells increased in synchronous G1 and S cells at 24 h after irradiation and began to resolve by 48 h (Figs 3B and 5B). Meanwhile, relative mtDNAcn was higher at 48 h than at 24 h in synchronous G1 cells, whereas it remained at a similar level between 24 and 48 h in synchronous S cells (Figs 3A and 5A). These findings suggest that the observed changes in relative mtDNAcn are not solely attributable to prolonged G2 arrest or delayed cell cycle progression, but might instead reflect a cell cycle- and dose-dependent enhancement of mitochondrial DNA synthesis in response to irradiation. However, the underlying mechanisms remain to be elucidated.

To achieve a higher degree of cell cycle synchronization, HeLa-FUCCI cells were treated with etoposide in the present study. Under non-cytotoxic conditions, this treatment effectively induced G2 phase arrest. However, the relative mtDNAcn dynamics observed under the conditions differed substantially from those obtained using the mitotic harvesting method. This discrepancy might reflect etoposide-induced alterations in mitochondrial physiology. In the present study, an increase in mtDNA copy number was observed 6 h after X-ray irradiation in HeLa-FUCCI cells pretreated with 1 μM etoposide for 24 h. This is consistent with a previous report in which etoposide promoted mitochondrial biogenesis through the ATM–AMPK signaling pathway in response to DNA damage in HeLa cells (Fu et al., 2008). In contrast, at 24 and 48 h after irradiation, a decrease in mtDNAcn was observed regardless of radiation dose. A similar decrease has been reported in HeLa cells treated with another topoisomerase inhibitor, ciprofloxacin, which significantly reduced mtDNA copy number after 3 days exposure by impairing mtDNA replication initiation and transcription (Hangas et al., 2018). Although the specific effects of etoposide on mitochondrial function remain to be elucidated, these findings suggest that drug-free synchronization methods, such as the mitotic harvesting method used in this study, might be advantageous for evaluating radiation-induced, cell cycle–dependent changes in mtDNA copy number without the confounding effects of drug-induced mitochondrial perturbation.

Importantly, although our current study is observational in nature, the data presented here raise testable hypotheses regarding the role of mtDNA content in modulating radiation responses. For example, increased mtDNA levels might support mitochondrial biogenesis and energy demands required for DNA repair, whereas depletion might impair cellular recovery. Future studies that directly manipulate mtDNA content or mitochondrial activity in synchronized cells at specific cycle phases will be crucial in elucidating these mechanisms.

### Technical limitations and future perspectives

Although this study provides novel insight into mtDNA copy number dynamics in relation to the cell cycle and radiation exposure, it remains exploratory and hypothesis generating. Several technical limitations should be acknowledged more explicitly. These include: (1) the partial synchronization of cell populations using mitotic harvesting, which results in mixed-phase cell populations; (2) the limited phase resolution of the FUCCI system, which does not allow strict cell cycle classification based on fluorescence alone; and (3) the use of genetically and chromosomally unstable HeLa cells, which could limit the generalizability of the findings. Owing to the diversity in the length of the G1 phase of each cell after mitosis (Shimono et al., 2020), the synchronization often breaks down after approximately one iteration of the cell cycle. We have attempted to mitigate these issues by validating synchronization with etoposide-induced G2 arrest. However, drug-induced synchronization introduces its own confounding factors. Future studies using more precise synchronization systems, such as FUCCI2 (Sakaue-Sawano et al., 2011) or FUCCI(CA) (Sakaue-Sawano et al., 2017), will be essential to validate the present observations and to investigate the mechanistic links between mtDNA dynamics and cell survival following irradiation. In addition, due to the aneuploidy of chromosomes in HeLa cells (Landry et al., 2013), the behavior of relative mtDNAcn might differ from that of normal cells. Thus, the cell cycle dependence might need to be verified in noncancerous cell lines such as RPE-1 FUCCI2, as the next step, although HeLa-FUCCI cells have been widely used to date as a model for cell cycle research (Sakaue-Sawano and Miyawaki, 2014).

It is well-known that cell survival following irradiation is influenced by the cell cycle phase at the time of exposure. A previous study reported that mitochondrial DNA (mtDNA) depletion alters γ-H2AX levels and cell viability in a cell type-dependent manner (Van Gisbergen et al., 2017), suggesting that mtDNA content modulates the DNA damage response and radiosensitivity via mechanisms involving oxidative stress and energy metabolism. In the present study, we found that the amount of mtDNA varies depending on the cell cycle phase at the time of irradiation. This observation, that the amount of mtDNA varies depending on the cell cycle phase at the time of irradiation, raises the possibility that mtDNA levels might be involved in the cell cycle-dependent differences in radiosensitivity. Although a causal relationship remains to be established, further studies incorporating direct functional assays will help clarify whether and how mtDNA content contributes to radiation response across different cell cycle phases.

## MATERIALS AND METHODS

### Cell culture

HeLa-FUCCI cells (RCB2812, provided by the RIKEN BioResearch Resource Center in Japan) were cultivated in Dulbecco's modified Eagle's medium (low glucose) (Gibco, Carlsbad, CA, USA), supplemented with 10% fetal bovine serum (Equitech-Bio Inc., Kerrville, TX, USA) and 1% penicillin-streptomycin (Sigma, St. Louis, MO, USA). The doubling time was ∼18–20 h, and the cells were passaged to maintain a confluence rate of 20–80%. These cell cultures were maintained in a humidified incubator at 37°C in a 95% air and 5% CO_2_ atmosphere.

### Mitotic harvesting method

Cultured animal cells that normally grow by adhering to surfaces often become rounded during mitosis, resulting in much weaker adhesion to the surface. This weak adhesion can be used to collect mitotic cells selectively to obtain a cell cycle-synchronous cell population while maintaining physiological conditions (Terasima and Tolmach, 1963b). In this study, we changed the medium before collecting the mitotic cells to remove dead cells. Next, the bottom of the dish in which the cells were cultured was tapped lightly, and the mitotic cells that were thinly adherent to the bottom were suspended in the medium. The medium containing the mitotic cells was collected and seeded into another dish (Fig. 1A). Fluorescence microscopy was used to check the fluorescence color in the nuclei of the cells and confirm the phase of the cell cycle.

### Etoposide treatment

Etoposide increases topoisomerase II (Topo II)-mediated DNA breakage primarily by inhibiting the religation of cleaved nucleic acid strands (Hande, 1998). Treatment with etoposide at concentrations of 0.1 μM or higher has been reported to produce a cell population highly synchronized in the G2 phase (Sakaue-Sawano et al., 2011). In this study, the culture medium was replaced with medium containing 1 μM etoposide (E1383, Sigma-Aldrich), and the cells were incubated for 24 h. After treatment, the cells were gently washed with PBS and then cultured in fresh medium. The cell cycle phase was confirmed based on the fluorescence color of the cell nuclei using a fluorescence microscope.

### Irradiation setting

Cells were plated and allowed to adhere overnight. The seeding density was 1.0×10^5^ cells/dish for HeLa-FUCCI cells. Cells were irradiated using a 150 kVp X-ray beam produced by an X-ray generator filtered through a 1.0 mm aluminium filter (MBR-1520R-4, Hitachi Power Solutions, Ibaraki, Japan) operating at a dose rate of 1.83 Gy/min. During irradiation, the absorbed dose in the air was monitored using an ionization chamber placed adjacent to the samples. The irradiation doses were 0 (control), 0.5, 2, 4 and 8 Gy, based on a previous study investigating the cell cycle dependence of cell survival in HeLa-FUCCI cells exposed to X-rays (Seino et al., 2024). Cells were cultured for up to 48 h after irradiation.

### Quantification of mitochondrial DNA copy numbers

To determine the mtDNAcn from the DNA samples, we used the Human Mitochondrial DNA Monitoring Primer Set (cat. #7246) and TB Green® Premix Ex Taq™ II (Tli RNase H Plus), 5×5 ml, ROX Plus (cat. #RR82WR) (Takara BIO Inc, Shiga, Japan). With the Applied Biosystem StepOne real-time PCR analysis (Thermo Fisher Scientific Inc., MA, USA), the thermal profile was as follows: 2 min at 98°C, 40 cycles at 98°C for 10 s, 15 s at 60°C, and 30 s at 68°C. The Ct values were automatically calculated by the Applied Biosystem software. The Ct values obtained from real-time PCR analysis are presented in Tables S1–S5.

To measure mtDNAcn relative to nDNA (Rooney et al., 2015), the primer set contained two primer pairs each for detecting mtDNA and nDNA, for a total of four primers (Fig. S1). These primers target two genes on mtDNA (*MT-ND1* and *MT-ND5*) and two genes on nDNA (*SLCO2B1* and *SERPINA1*). To determine the relative mtDNAcn, first, the difference in Ct values of the *MT-ND1* and *SLCO2B1* pair was measured and then ΔCt1 (Ct value of *SLCO2B1*−Ct value of *ND1*) was calculated. Similarly, the difference in Ct values for the *MT-ND5* and *SERPINA1* pair was measured and ΔCt2 (Ct value of *SERPINA1*−Ct value of *ND5*) was calculated. Finally, 2N (*N*=ΔCt) was obtained from the ΔCt1 and ΔCt2 values and the average of these two values was used as the copy number.

### Clonogenic assay

The effect of etoposide on radiosensitivity was assessed using a clonogenic assay. HeLa-FUCCI cells cultured in 150 mm dishes were treated with 1 μM etoposide for 24 h and then irradiated with X-rays at room temperature. Immediately after irradiation, appropriate numbers of cells were trypsinized and replated onto new 150 mm dishes at low density. The cells were cultured for ∼14 days to allow colony (≥50 cells) formation. Colonies were fixed and stained with Giemsa stain (Merck, Darmstadt, Germany), and the number of colonies was counted manually.

The plating efficiency (PE) was calculated as the ratio of the number of colonies formed to the number of cells seeded. The surviving fraction at each radiation dose was obtained by normalizing the PE to that of the non-irradiated control. Survival curves were fitted using the linear-quadratic (LQ) model, which describes two components of radiation-induced cell death: a linear component proportional to dose (αD) and a quadratic component proportional to the square of the dose (βD²) (Steel and Peacock, 1989).

### Statistical analysis

Each experiment was performed independently at least three times. The Pearson's correlation coefficient was employed to evaluate the correlations between the culture time and the mtDNAcn. The chi-squared test was used to compare the ratios of cell cycle phases. Statistical significance was set at *P*<0.05 on both sides for a continuous model. In addition, to account for the increased possibility of type-I errors due to multiple testing, we used the Bonferroni correction to adjust the significance level. Python Anaconda version 23.7.4 was used for all statistical analyses.

## Supplementary Material

10.1242/joces.263642_sup1 Supplementary information
