# Supplementary material for: Cell cycle- and dose-dependent effects on mitochondrial DNA copy number variation following irradiation
Source: J Cell Sci. 2025 Aug 8;138(15):jcs263642. doi: 10.1242/jcs.263642 (PMC12377708; doi:10.1242/jcs.263642)
Supplement: Supplementary information [file joces-138-263642-s1.pdf]

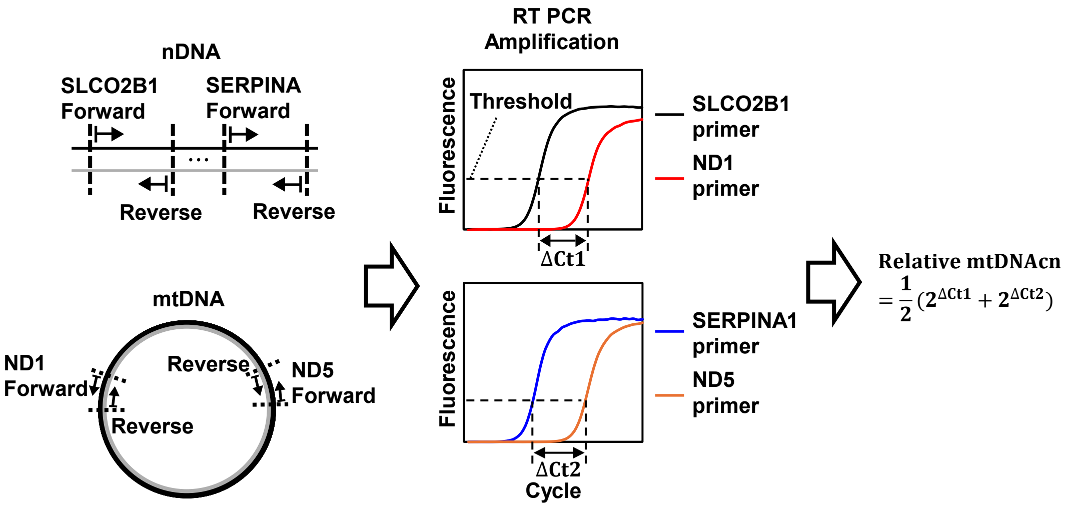

Fig. S1. Estimation of mtDNA/nDNA ratio using real-time PCR.

The mtDNA/nDNA ratio was estimated using two primer sets: SLCO2B1 and SERPINA for nDNA, and ND1 and ND5 for mtDNA. Then, the relative mtDNAcn was calculated as the average of $2^{\Delta Ct1}$ and $2^{\Delta Ct2}$ where ΔCt1 is the difference in Ct values between the SLCO2B1 and ND1 primers, and ΔCt2 is the difference in Ct values between the SERPINA and ND5 primers.

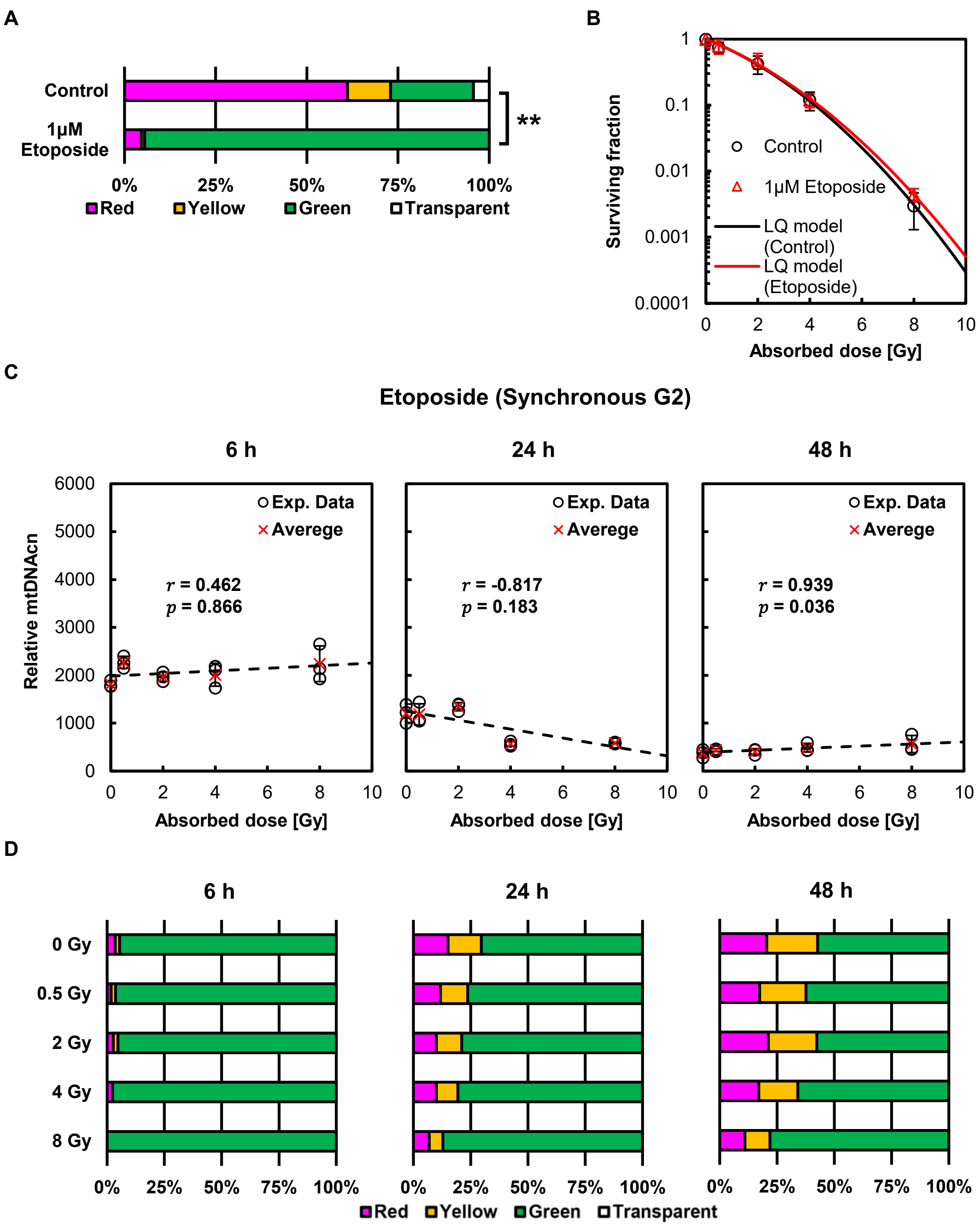

Fig. S2. Post-exposure relative mtDNAcn changes with etoposide treatment.

(A) Nuclear fluorescence distribution after treatment with 1 μM etoposide for 24 hours. Compared to untreated controls, the proportion of green fluorescence (indicative of G2 phase) significantly increased (χ^2 test, $^{**}p < 0.001$), confirming effective G2 synchronization.

Journal of Cell Science • Supplementary information

(B) Clonogenic survival of HeLa-FUCCI cells pretreated with or without 1 µM etoposide. Cells were irradiated with X-rays and cultured for 14 days, followed by Giemsa staining. Survival curves were fitted using the linear-quadratic (LQ) model. No significant difference in radiosensitivity was observed between control and etoposide-treated cells.

(C) Temporal changes in relative mtDNAcn following irradiation in etoposide-treated cells. A marked increase in mtDNAcn was observed at 6 hours post-irradiation across all doses, followed by a decline at 24 hours and a further decrease at 48 hours.

(D) Nuclear fluorescence profiles following irradiation in etoposide-treated cells. No statistically significant differences in fluorescence distribution were observed among the irradiated groups compared to the non-irradiated group (χ^2 test, $p > 0.05$ for all doses).

Table S1. Real-time PCR Ct values from cell cycle-synchronized cells obtained via mitotic harvesting method.

A

Time [h]	Ct value ND1	Ct value SLCO2B1	Ct value ND5	Ct value SERPINA	Relative mtDNAcn
0	19.56	29.74	18.61	29.83	1775
2	16.96	26.56	15.96	26.62	1197
4	17.75	28.01	16.78	27.97	1781
6	19.66	29.76	18.53	30.06	2033
8	17.81	27.47	16.80	27.74	1386
10	14.74	24.91	13.76	24.93	1733
12	19.11	29.57	18.16	29.55	2044
14	15.88	25.43	14.92	25.46	1119
16	14.94	24.86	13.96	24.76	1372
18	16.87	26.89	15.87	26.89	1556
20	14.95	24.92	13.86	24.88	1542
22	13.93	24.69	12.94	24.41	2291
24	19.19	28.43	17.74	28.94	1476

B

Time [h]	Ct value ND1	Ct value SLCO2B1	Ct value ND5	Ct value SERPINA	Relative mtDNAcn
0	14.82	26.00	14.36	25.43	2236
2	14.70	25.52	14.99	25.33	1546
4	15.66	25.72	14.92	25.56	1334
6	15.38	26.29	14.67	25.88	2151
8	15.51	26.58	14.72	26.41	2724
10	15.46	25.76	14.89	25.74	1552
12	15.76	26.25	15.22	26.12	1671
14	15.97	26.23	15.26	26.41	1743
16	16.22	26.32	15.53	26.20	1366
18	15.64	26.02	14.95	25.94	1677
20	15.45	26.70	14.79	25.93	2351
22	15.93	26.38	15.24	26.70	2114
24	16.21	27.25	15.49	27.14	2657

C

Time [h]	Ct value ND1	Ct value SLCO2B1	Ct value ND5	Ct value SERPINA	Relative mtDNAcn
0	15.01	26.32	14.68	25.58	2228
2	15.38	26.08	14.95	25.69	1691
4	14.99	25.71	14.53	25.14	1628
6	15.45	26.62	15.08	26.06	2160
8	15.34	26.62	14.89	26.28	2581
10	14.96	25.75	14.60	25.54	1861
12	15.42	26.52	15.12	25.87	1958
14	15.55	25.96	15.08	25.57	1399
16	16.32	26.51	15.98	26.05	1120
18	14.94	25.93	14.58	25.48	1968
20	15.35	26.45	14.93	25.96	2140
22	15.63	27.16	15.20	26.73	2956
24	15.94	26.75	15.46	26.38	1862

Ct values obtained by real-time PCR analysis of DNA extracted every 2 hours up to 24 hours after the mitotic harvesting method was applied. The table presents the Ct values for mtDNA (MD1 and MD5) and nDNA (SLCO2B1 and SERPINA) from three independent experiments (A-C).

Journal of Cell Science • Supplementary information

Table S2. Real-time PCR Ct values for the nuclei of G1 phase cells after X-ray irradiation.

A

Time [h]	Absorbed dose [Gy]	Ct value ND1	Ct value SLCO2B1	Ct value ND5	Ct value SERPINA	Relative mtDNAcn
6	0	19.34	29.12	18.31	28.61	1068
	0.5	20.13	29.80	18.93	29.18	1015
	2	19.41	29.15	18.40	28.78	1096
	4	19.71	29.01	18.83	29.03	904
	8	19.15	28.87	18.15	28.41	1036
24	0	18.87	28.93	17.96	27.93	1038
	0.5	19.22	29.10	18.26	28.67	1148
	2	19.63	29.88	18.49	29.37	1547
	4	19.13	29.57	18.83	29.40	1455
	8	19.35	29.76	18.67	29.27	1461
48	0	19.18	29.91	19.10	28.74	1249
	0.5	19.02	29.67	18.26	28.92	1615
	2	19.06	29.70	18.13	28.98	1725
	4	19.26	29.32	18.33	28.98	1333
	8	19.06	30.95	18.64	31.13	4762

B

Time [h]	Absorbed dose [Gy]	Ct value ND1	Ct value SLCO2B1	Ct value ND5	Ct value SERPINA	Relative mtDNAcn
6	0	18.92	29.13	18.52	28.52	1106
	0.5	18.92	28.68	18.30	28.59	1060
	2	19.45	28.55	18.70	28.76	806
	4	18.96	28.77	18.51	28.93	1136
	8	19.70	29.41	18.98	29.32	1068
24	0	18.57	28.40	18.04	27.97	941
	0.5	19.05	29.77	18.50	29.14	1643
	2	18.87	29.02	18.50	28.92	1255
	4	19.36	29.85	18.56	29.49	1697
	8	19.22	29.92	18.31	29.40	1926
48	0	18.55	29.11	18.22	28.93	1594
	0.5	18.04	29.64	18.28	29.47	2719
	2	18.46	29.56	18.12	29.25	2219
	4	18.56	29.50	17.93	29.10	2134
	8	18.97	30.44	18.45	30.40	3409

C

Time [h]	Absorbed dose [Gy]	Ct value ND1	Ct value SLCO2B1	Ct value ND5	Ct value SERPINA	Relative mtDNAcn
6	0	19.38	29.09	18.92	29.23	1052
	0.5	19.21	28.93	18.50	28.98	1135
	2	18.96	29.14	18.47	28.98	1311
	4	19.60	28.98	19.15	29.36	927
	8	19.52	29.15	18.76	29.22	1101
24	0	18.92	29.18	18.42	28.96	1361
	0.5	19.10	29.93	18.57	29.51	1893
	2	18.69	29.43	18.32	29.23	1821
	4	18.26	29.14	18.38	29.34	1936
	8	19.02	29.73	18.70	29.68	1847
48	0	18.75	29.28	18.45	29.01	1490
	0.5	18.88	29.57	18.43	29.61	1988
	2	18.98	29.93	18.46	29.51	2050
	4	18.32	29.33	18.23	29.47	2242
	8	18.83	30.98	18.22	30.97	5730

Journal of Cell Science • Supplementary information

Table S3. Real-time PCR Ct values for the nuclei of G2 phase cells after X-ray irradiation.

A

Time [h]	Absorbed dose [Gy]	Ct value ND1	Ct value SLCO2B1	Ct value ND5	Ct value SERPINA	Relative mtDNAcn
6	0	17.79	27.83	17.18	28.08	1485
	0.5	17.93	27.79	16.97	27.36	1136
	2	17.91	27.98	17.30	27.59	1165
	4	17.51	28.02	17.19	27.96	1601
	8	17.53	28.08	16.99	27.57	1515
24	0	15.97	26.92	15.48	26.67	2161
	0.5	16.03	26.44	15.53	26.36	1592
	2	15.89	26.47	15.43	26.38	1757
	4	16.08	26.93	15.63	26.93	2182
	8	16.59	27.98	15.92	27.92	3393
48	0	17.38	27.76	17.06	27.64	1429
	0.5	17.71	27.97	17.24	27.95	1455
	2	17.66	27.95	17.32	27.86	1372
	4	17.72	27.96	17.51	27.93	1289
	8	17.66	27.63	16.94	27.59	1307

B

Time [h]	Absorbed dose [Gy]	Ct value ND1	Ct value SLCO2B1	Ct value ND5	Ct value SERPINA	Relative mtDNAcn
6	0	17.49	28.22	16.76	27.73	1851
	0.5	17.29	28.09	16.94	27.87	1863
	2	17.48	27.96	16.99	27.17	1297
	4	17.33	28.15	16.92	27.42	1630
	8	17.56	28.21	17.11	27.58	1515
24	0	15.93	26.77	15.50	26.32	1815
	0.5	16.06	26.69	15.62	26.07	1491
	2	16.14	27.09	15.61	26.47	1918
	4	16.02	26.97	15.77	26.63	1923
	8	16.55	28.39	15.98	27.55	3347
48	0	18.24	28.43	17.80	27.63	1042
	0.5	18.07	28.32	17.54	27.93	1282
	2	17.73	28.11	17.23	27.69	1365
	4	17.54	27.99	17.20	27.55	1348
	8	17.26	28.21	16.82	27.67	1909

C

Time [h]	Absorbed dose [Gy]	Ct value ND1	Ct value SLCO2B1	Ct value ND5	Ct value SERPINA	Relative mtDNAcn
6	0	17.45	27.78	16.86	27.48	1429
	0.5	17.93	28.09	17.19	27.65	1276
	2	17.98	28.21	17.00	27.50	1323
	4	17.82	28.13	17.07	27.92	1559
	8	17.92	28.49	17.13	28.07	1739
24	0	16.92	27.40	15.92	26.90	1724
	0.5	16.25	27.39	15.48	26.83	2435
	2	16.15	27.15	15.43	26.86	2402
	4	16.28	27.30	15.58	27.13	2531
	8	17.28	28.92	16.58	28.62	3700
48	0	18.31	28.54	17.67	27.77	1149
	0.5	18.30	28.74	17.52	28.25	1543
	2	18.13	28.77	17.32	28.29	1800
	4	17.80	28.65	17.48	27.93	1623
	8	17.87	28.41	17.20	28.33	1869

Table S4. Real-time PCR Ct values for the nuclei of S phase cells after X-ray irradiation.

A

Time [h]	Absorbed dose [Gy]	Ct value ND1	Ct value SLCO2B1	Ct value ND5	Ct value SERPINA	Relative mtDNAcn
6	0	16.19	25.93	14.92	25.82	1388
	0.5	17.57	28.42	17.71	27.80	1472
	2	17.75	27.71	17.15	27.46	1138
	4	17.93	27.96	17.51	27.75	1126
	8	17.84	28.13	17.36	27.76	1301
24	0	17.62	28.22	17.17	27.61	1469
	0.5	17.65	28.14	17.27	27.86	1492
	2	17.40	28.11	16.88	27.58	1671
	4	17.41	28.35	16.96	27.93	1983
	8	17.32	28.92	16.96	28.30	2857
48	0	16.86	27.54	16.42	27.33	1782
	0.5	17.07	27.72	16.60	27.40	1698
	2	17.46	28.19	16.95	27.93	1855
	4	17.08	27.93	16.63	27.42	1810
	8	17.03	28.43	16.66	28.04	2679

B

Time [h]	Absorbed dose [Gy]	Ct value ND1	Ct value SLCO2B1	Ct value ND5	Ct value SERPINA	Relative mtDNAcn
6	0	17.37	27.45	16.86	26.72	1009
	0.5	17.43	27.43	16.91	26.71	958
	2	17.93	27.97	17.32	27.09	966
	4	17.80	27.97	17.31	27.39	1116
	8	17.74	27.95	17.16	27.37	1187
24	0	17.29	27.85	16.88	26.97	1299
	0.5	17.46	27.78	16.93	27.03	1189
	2	17.26	28.20	16.67	27.19	1717
	4	17.27	28.27	16.85	27.71	1956
	8	17.40	28.70	16.76	27.83	2335
48	0	16.60	27.21	16.11	26.59	1498
	0.5	16.86	27.38	16.24	26.93	1560
	2	17.11	27.91	16.67	27.34	1705
	4	16.64	27.65	16.20	27.00	1922
	8	16.99	28.12	16.41	27.64	2327

C

Time [h]	Absorbed dose [Gy]	Ct value ND1	Ct value SLCO2B1	Ct value ND5	Ct value SERPINA	Relative mtDNAcn
6	0	16.86	26.79	16.65	26.66	1004
	0.5	17.29	26.95	16.98	26.88	881
	2	17.74	28.02	17.30	27.25	1117
	4	17.70	27.95	17.42	27.49	1144
	8	17.78	28.01	17.49	27.54	1129
24	0	17.35	27.91	16.93	27.18	1368
	0.5	17.14	27.96	16.89	27.17	1522
	2	17.11	28.00	16.79	27.38	1723
	4	17.19	28.13	16.93	27.60	1801
	8	17.43	28.57	17.03	27.84	2027
48	0	16.60	27.10	16.36	26.43	1262
	0.5	16.93	27.42	16.55	26.93	1382
	2	16.79	27.63	16.49	26.98	1629
	4	16.78	27.92	16.97	27.63	1932
	8	16.79	27.93	16.45	27.54	2220

Journal of Cell Science • Supplementary information

Table S5. Real-time PCR Ct values for the nuclei of etoposide-treated cells after X-ray irradiation.

A

Time [h]	Absorbed dose [Gy]	Ct value ND1	Ct value SLCO2B1	Ct value ND5	Ct value SERPINA	Relative mtDNAcn
6	0	16.04	26.78	15.66	26.51	1783
	0.5	15.97	27.03	15.55	26.77	2257
	2	16.07	27.07	15.60	26.63	2066
	4	15.98	26.93	15.63	26.77	2112
	8	16.03	26.97	15.89	26.78	1930
24	0	14.91	25.10	14.61	24.93	1226
	0.5	14.67	25.02	14.43	25.05	1439
	2	15.69	25.95	15.34	25.64	1246
	4	15.83	25.47	14.93	22.87	522
	8	15.67	25.51	14.71	22.75	587
48	0	16.39	25.65	15.47	22.74	386
	0.5	16.27	25.79	15.22	22.77	461
	2	16.19	25.68	15.47	22.97	448
	4	16.42	25.97	15.64	22.64	440
	8	16.18	26.76	21.73	22.93	771

B

Time [h]	Absorbed dose [Gy]	Ct value ND1	Ct value SLCO2B1	Ct value ND5	Ct value SERPINA	Relative mtDNAcn
6	0	15.65	26.46	15.36	26.12	1769
	0.5	16.05	27.25	15.72	26.98	2403
	2	15.67	26.66	15.41	26.16	1874
	4	15.43	25.95	14.88	25.85	1739
	8	15.94	26.90	15.59	26.75	2139
24	0	14.93	24.77	14.55	24.64	1004
	0.5	15.06	24.87	14.56	24.84	1068
	2	15.47	25.93	15.27	25.71	1401
	4	15.78	25.57	14.95	22.82	559
	8	15.74	25.55	14.86	22.77	569
48	0	17.08	25.93	16.26	22.82	278
	0.5	16.15	25.62	15.32	22.89	449
	2	16.38	25.83	15.52	22.96	437
	4	16.43	25.98	15.60	22.50	434
	8	16.18	25.73	15.39	22.92	469

C

Time [h]	Absorbed dose [Gy]	Ct value ND1	Ct value SLCO2B1	Ct value ND5	Ct value SERPINA	Relative mtDNAcn
6	0	15.68	26.58	15.34	26.21	1895
	0.5	15.95	26.91	15.62	26.80	2153
	2	15.61	26.63	15.25	26.12	1970
	4	16.06	27.19	15.78	26.84	2185
	8	15.93	27.27	15.65	27.05	2654
24	0	15.27	25.50	14.93	25.55	1390
	0.5	15.04	25.00	14.90	25.00	1047
	2	15.61	25.91	15.33	25.89	1387
	4	15.34	25.23	14.55	22.83	628
	8	15.69	25.58	14.80	22.83	604
48	0	16.26	25.78	15.44	22.80	450
	0.5	16.27	25.66	15.58	22.78	409
	2	16.93	26.05	16.03	22.91	337
	4	15.56	25.40	14.76	22.83	593
	8	16.26	25.75	15.39	22.95	453

Journal of Cell Science • Supplementary information
